# Motor planning, initiation and execution of shoulder abduction against gravity: Evidence from startReact

**DOI:** 10.1371/journal.pone.0346615

**Published:** 2026-04-10

**Authors:** Christina Thomas, Brianna Johnson, Emma M. Baillargeon, Rosalind L. Heckman

**Affiliations:** 1 Department of Physical Therapy, School of Pharmacy and Health Professions, Creighton University, Omaha, Nebraska, United States of America; 2 Department of Physical Therapy and Human Movement Sciences, Feinberg School of Medicine, Northwestern University, Chicago, Illinois, United States of America; Drexel University School of Biomedical Engineering Science and Health Systems, UNITED STATES OF AMERICA

## Abstract

Stroke affects the sensorimotor control of the upper extremity, limiting functional movements which require coordination between the shoulder and distal joints. StartReact has been used to investigate motor planning, independent of motor initiation and execution, but it is unclear if observed deficits in motor planning at the shoulder are due to task requirements or stroke-related impairments. Our aim was to decouple these factors by studying motor planning of shoulder movement in participants who have not had a stroke. Participants performed unilateral shoulder abduction movements from two initial positions in response to auditory cues. Muscle activity was recorded from the sternocleidomastoid (SCM) muscles as an indicator of startReact. Middle deltoid (DELT) activity and shoulder abduction position were also recorded. We hypothesized that unilateral shoulder abduction could be triggered by a loud acoustic stimulus (LAS) used to study startReact. We further hypothesized that these LAS-triggered movements would be initiated at shorter onset latencies but otherwise would not differ in movement timing or kinematics compared to volitional movements as evidence of motor planning. We found a significantly higher probability of early SCM activity with LAS delivery, consistent with evidence of motor planning for unilateral shoulder abduction from both initial positions. Shoulder abduction onset, movement duration, and DELT onset were shorter and peak velocity was greater with LAS-triggered movement compared to voluntary movement for both initial positions. Differences in the displacement between LAS-triggered and voluntary movements depended on task training. Overall, our findings further knowledge of shoulder sensorimotor control and highlight challenges with using startReact to investigate stroke-related movement impairments.

## Introduction

Stroke affects sensorimotor control of reaching across the phases of movement, from conception to termination [1,2], and complete functional recovery is often limited [3,4]. Stroke rehabilitation and research have documented impairments in motor execution, including decreased movement speed [5,6] and abnormal coupling across the upper extremity [7–10]. However, motor planning and initiation [11–15] are largely unobservable, limiting their clinical assessment and intervention. StartReact, a method more commonly used in research, has been used to overcome this challenge and investigate motor planning independent of motor initiation and execution.

The startReact methodology delivers a high intensity, potentially startling, loud acoustic stimulus (LAS) to trigger the involuntary release of a motor action if an action has been prepared. StartReact can then be used to dissociate the planning and initiation phases of movement and allow observation of the prepared motor action [16]. StartReact responses have been considered evidence of motor planning if there is a significant reduction in the latency of muscle and movement onset compared to voluntary responses and if an independent indicator of a startle response is detected, typically activation of the sternocleidomastoid muscle (SCM) [17]. Some protocols have measured movement completion time and displacement as evidence that the prepared motor action was consistent with the instructed task whether initiated voluntarily or triggered involuntarily by a LAS [16–18]. Though startReact methods have been used to investigate the influence of stroke on motor planning [19–24], it remains unclear if and how motor planning deficits contribute to altered upper extremity movements in individuals following stroke.

There is mixed evidence for impairments in motor planning following a stroke. Using startReact, Honeycutt and Perreault showed that motor planning was intact for ballistic movement of the elbow with startle-triggered movement in the direction of instructed elbow flexion or extension [19]. Evidence from startReact has also suggested motor planning is intact following stroke for hand opening [21]. These findings motivate ongoing research to determine the therapeutic potential for exciting functional movement with startReact. However, the therapeutic potential of startReact may be limited to distal movements. Fewer startle-triggered movement responses have been reported for tasks that require shoulder activation [24], including reaching against gravity [22,23]. A recent study investigating bilateral shoulder abduction using startReact established motor planning in young adults without neurological deficits [25]. However, the 30-degree shoulder abduction movement investigated in the study was initiated with participants’ arms relaxed at their sides, requiring minimal movement against gravity. Therefore, it is unclear whether the impaired planning observed for unilateral reaching movements in prior studies post stroke is due to task differences (such as the influence of gravity) or due to impairments following stroke.

Our aim was to examine motor planning of shoulder movements against gravity in young adults without neurological deficits to understand the influence of gravity without the potentially confounding influence of stroke. We studied shoulder abduction as a uniplanar movement, similar to startReact protocols used at the elbow and wrist. We examined movement from two initial positions to compare the influence of gravity on the planning, initiation, and execution of shoulder abduction throughout the range of motion. To probe the presence of a prepared motor action, we delivered a LAS on a subset of movements and measured the resulting kinematics and muscle activity. We compared the initiation and execution of the motor action under volitional control to motor actions triggered by the LAS. We hypothesized that task initial position would not influence motor planning at the shoulder. Specifically, we expected that movements triggered by the LAS would occur at shorter onset latencies with consistent movement displacement compared to movements initiated voluntarily from either position. Finally, we completed a control experiment to understand the influence of our training protocol, and our findings suggest motor planning of shoulder abduction is influenced by training but not initial position.

## Methods

### Participants

Sixteen right-hand dominant individuals (9 female, age: 21 ± 3 years) with no self-reported history of neurological deficits, shoulder injury, or shoulder pain were recruited from a university campus (**Table in** S1 Table). Participants were excluded if they reported a sensitivity or history of adverse reaction to loud noise. The study was approved by the Creighton University Institutional Review Board (Protocol 2002507) and written informed consent was received from every participant. Participant recruitment was conducted from September 30, 2022–December 8, 2023. The participant pictured in this manuscript has given written informed consent (as outlined in PLOS consent form) to publish these details.

### Equipment and protocol

**Fig 1:**
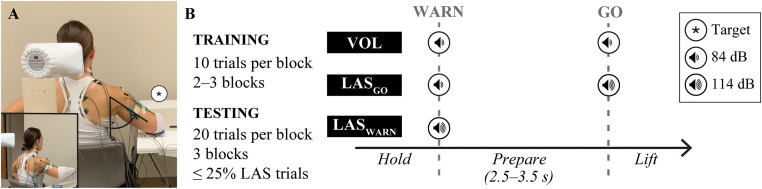
Participant setup and protocol. **(A)** Participants were seated with the forearm supported and shoulder abducted to the desired initial position (45° or 75°; 45° illustrated here) measured as the angle between the thorax and humerus. Participants abducted the shoulder 30° (inset) in response to auditory cues delivered according to the experimental protocol. **(B)** For each starting position, participants completed a training phase of voluntary trials and a testing phase with a LAS delivered ≤ 25% of trials. Auditory cues were delivered as WARN and GO cues with a randomized time between cues to allow sufficient time for motor planning, consistent with trial type (VOL, LAS_GO_, LAS_WARN_), and to reduce anticipation of the GO cue. dB: decibel, GO: cue to move, LAS: loud acoustic stimulus, s: seconds, VOL: voluntary, WARN: cue to prepare, °: angular degrees.

Our main objective was to investigate motor planning of unilateral, shoulder abduction movement performed against gravity. Participants were seated with back support inferior to the scapula next to a height-adjustable table with their right arm abducted in the frontal plane, elbow flexed to 90º, and forearm supported on the table (**Fig 1A**). Participants performed shoulder abduction by lifting the elbow as quickly as possible in response to auditory stimuli. A low-intensity acoustic stimulus of 84 dB was delivered with a duration of 40 ms to guide the preparation and execution of shoulder abduction movement (QSI-4310 Piezo Buzzer). The first auditory stimulus signaled the start of each trial (WARN) with instructions to prepare, and the second auditory stimulus cued the participant to execute a 30° ballistic shoulder abduction movement as quickly as possible (GO). The time between WARN and GO cues was randomly distributed between 2.5 and 3.5 s to limit anticipation of the GO cue and allow ample time for motor planning [26]. On a subset of trials, a potentially startling LAS was delivered using a piezo-dynamic loud horn (M85PDS; MG Electronics) to investigate the startle response and preparedness of a motor action, referred to as StartReact (**Fig 1B**). The loud horn was positioned behind the participant, in midline, at the height of the external acoustic meatus to achieve an acoustic stimulus intensity of 114 dB (1,745 Hz tone, impulse measurement) (PCE-428; PCE Instruments).

Two initial shoulder positions were used (45° and 75° abduction) to alter control of the humerus, including the influence of gravity and coordination of the scapulothoracic and glenohumeral joints of the shoulder complex [27,28]. Testing of each initial position was blocked, and the order was randomized across participants. Before each experimental block, the height of the table was adjusted to achieve the desired initial position as measured with a manual goniometer. For some participants, the range of table heights was insufficient to achieve the 45° initial position, and testing was completed with the smallest achievable shoulder abduction angle (**Table in** S1 Table).

A training phase was used for each initial position to familiarize participants with the ballistic 30° abduction movement task. Participants were instructed to focus on lifting their elbow off the table and to maintain the 30° target position briefly before returning to the table. Participants practiced moving as fast as possible in response to the GO cue. The 30° target position was measured with a manual goniometer and externally cued by the experimenter intermittently during training. Participants were instructed to fully relax between trials and maintain neutral trunk posture. For both initial positions, participants completed blocks of 10 training trials until they demonstrated consistent, rapid responses to the GO cue with approximately 30° of shoulder abduction over five consecutive trials [19,29,30]. The LAS was randomly delivered 1 or 2 times during the training phase to assess the participant’s baseline startle response. An average of 27 ± 4 (range: 19–34) training trials were completed across participants and initial positions; this amount of training is consistent with prior studies [20,30,31].

Following training, the testing phase for each initial position consisted of three blocks of 20 trials. To probe the presence of a prepared motor action, the LAS was delivered in place of the GO cue on 4 or 5 trials per testing block (LAS_GO_). The LAS was delivered in place of the WARN cue on 1–3 trial(s) per initial position to probe the response to the LAS in the absence of an instructed motor preparation (LAS_WARN_). For each participant and initial position, the LAS was delivered in 13–15 of 60 testing trials (≤25%) to avoid habituation, consistent with startReact protocols [18,19,29,30].

Arm position was recorded throughout the trials using two electrogoniometers (Biometrics, Ltd.). One electrogoniometer recorded shoulder abduction in the frontal plane. The proximal arm of the electrogoniometer was placed on the participant’s thorax, inferior to the posterior axillary fold and lateral to the lateral border of the scapula. The distal arm was placed on the posterior humerus in line with the olecranon process. A second electrogoniometer recorded elbow flexion and extension with the proximal arm of the electrogoniometer placed along the midline of the lateral humerus and the distal arm of the goniometer placed on the dorsal surface of the forearm in line with the 3^rd^ metacarpal. These landmarks were selected based on accepted practice for manual goniometer measurements [32] and adjusted to increase measurement accuracy based on the observed movement of the shoulder complex for each participant. Raw voltage measured from the electrogoniometers was calibrated to joint angle using the Biometrics, Ltd. DataLINK software to achieve a linear regression fit > 0.97 for all measured joints across participants.

Surface electromyography (EMG) was recorded from the right middle head of the deltoid (DELT) and left and right sternocleidomastoid neck (SCM) muscles using disposable, bipolar electrodes (Bagnoli-16 System; Delsys Inc.) and following established protocols [33]. EMG activity was amplified and bandpass filtered from 20 to 450 Hz. Electrogoniometer and EMG signals were sampled at 2,000 Hz (PCI-2515; Measurement Computing).

We recorded a maximum voluntary isometric contraction (MVC) for each muscle with each participant to normalize EMG signal amplitude during movement. MVCs were performed in a seated position with the back supported below the level of the inferior angle of the scapula. The MVC for the DELT was measured by instructing the participant to hold the position of 90° shoulder abduction and 90° elbow flexion against resistance applied just proximal to the elbow [32]. The MVC for each SCM muscle was measured with contralateral rotation and ipsilateral flexion of the neck to the testing side [32]. Resistance was applied to the frontal bone above the ipsilateral eye. Participants practiced each movement prior to recording two repetitions with verbal encouragement for maximal effort consistent with previous protocols [34,35]. Participants rested a minimum of 30 seconds between repetitions. The maximum detrended, rectified EMG recorded over 0.5 seconds during MVCs was used to scale the amplitude of the EMG for each muscle.

Thoracic posture has been shown to influence scapular position and shoulder complex musculature [36], so we quantified each participant’s standing thoracic posture using the occiput-to-wall distance (OWD) [37] to account for possible individual anatomical differences in scapular motion. The OWD was measured using the two-ruler method and an average of three measurements [37]. Participants were cued to stand against the wall without shoes, with the inferior orbital margin and superior acoustic meatus passively positioned in the transverse plane parallel to ground. Since only one participant had OWD indicative of static thoracic hyperkyphosis (> 6.5 cm) (**Table in** S1 Table) [37], we were unable to investigate the influence of thoracic posture on the phases of shoulder abduction movement.

### Data and statistical analyses

We quantified initiation (movement and muscle onset latency) and execution (movement duration, displacement, velocity) of each shoulder abduction movement using kinematic and EMG measures. First, the angular velocity of shoulder abduction was calculated from the shoulder abduction angle measured by the electrogoniometer. The onset latency of shoulder abduction movement was defined as when the velocity exceeded 6°/s following the GO cue. The offset latency of shoulder abduction movement was defined as when the velocity returned below 2°/s, and movement duration was calculated as the difference between offset and onset latencies.

The velocity thresholds were selected to differentiate the ballistic shoulder abduction movement from volitional adjustments of initial posture or end point position. Displacement was calculated as the difference in shoulder abduction angle between movement offset and the mean initial position measured 100–200 ms before the GO cue or LAS. To investigate coordinated joint movement, elbow displacement was also calculated as the change in elbow angle from initial position to movement offset. Finally, peak shoulder abduction velocity was measured as the maximum velocity between movement onset and offset. Kinematic measurements were manually reviewed by one experimenter (BJ) to ensure movement onset corresponded to initiation of the ballistic movement and not superfluous non-task-related movements. The reviewer was blind to trial type.

EMG recordings for the DELT and SCM were processed to identify the onset of muscle activity. EMG signals were rectified after subtracting the mean recorded for each trial. The rectified EMG recordings were normalized by the average rectified EMG recorded over a 0.5 second window during MVCs for each muscle. Background muscle activity was calculated as the mean from 0 to 100 ms before delivery of the GO cue or LAS. DELT and SCM muscle onset latencies for each trial were first determined by identifying when EMG signals filtered using a low-pass Butterworth filter with a cut-off frequency of 50 Hz exceeded a threshold two standard deviations above the mean background muscle activity [26,29,38]. One experimenter (CT) then reviewed EMG recordings and onset latencies for all trials and manually adjusted onset latencies to the initial rise of unfiltered EMG above background activity when indicated [19,25,39]. Manual adjustments of muscle onset latencies were needed to account for trials with EMG artifact related to the cardiac cycle or tonic muscle activation. When manual adjustments were not sufficient to clearly identify muscle onset due to EMG artifact, the trial was excluded. During EMG review, trials were presented in a random order, and the experimenter was blind to trial type.

To describe motor planning, we detected the presence of a prepared motor action using early activation of the SCM [16] and described the response to the LAS. Trials with either right or left SCM muscle activation within 120 ms of the GO or LAS, a traditionally used cutoff latency [17,19,20,31,39–41], were classified as SCM + . We then quantified the proportion of SCM+ responses for voluntary (low intensity GO cue) and LAS_GO_ trials. Two participants had SCM+ responses for <10% of LAS_GO_ trials and were excluded from analysis as low startlers, consistent with prior studies [29,30,42]. These two participants also did not demonstrate a baseline startle response to the LAS delivered at rest during training as evidenced by absent or delayed SCM muscle activation. Though voluntary trials with SCM+ responses have traditionally been infrequent and excluded from analysis [20,21], we included them here because the anatomy of the SCM muscle allows for contribution to the control of proximal arm movement.

Experimental trials were reviewed for inclusion in data analysis. Voluntary trials with ballistic movement in response to the GO cue were included for analysis. Across all participants, < 4% of voluntary trials were excluded for no, early, or late movement. Trials were considered to have no movement if velocity did not exceed 6°/s (3 trials). Trials were excluded for early movement, unlikely to have occurred in response to the auditory cues, if the onset time was less than two standard deviations of the participant’s average voluntary movement onset time (11 trials). Trials were excluded for late movement, likely to have resulted from decreased attention to the task, if the onset time was more than two standard deviations of the participant’s average voluntary movement onset time (40 trials). LAS_GO_ trials were only excluded from analysis if the movement onset time was less than 50 ms, as movement was likely to have been initiated prior to delivery of the LAS (1 trial, < 1%). LAS_GO_ trials with no movement and late movement responses were included to quantify the probability that a motor action was prepared. Finally, 15 trials (<1%) were excluded by the experimenter during testing due to protocol errors (participant talking, moved multiple times, etc.) or during analysis due to poor signal quality. Across participants and trial types, 4.0 ± 1.9% of testing trials were excluded from analysis.

We performed an a priori power analysis to inform our sample size based on prior StartReact studies. We considered that for adults without a history of stroke, the onset latency of the agonist muscle during an elbow extension movement was reduced by approximately 50 ms when triggered by a LAS and estimated the standard deviation of onset latency as 36 and 14 ms for voluntary and LAS-triggered movements, respectively [19]. A sample size of five participants was determined necessary to achieve an effect size > 1.0 for a power level of 0.80 between LAS-triggered and voluntarily initiated movement onset latencies (G*Power, http://www.gpower.hhu.de/). Since approximately 15% of individuals do not respond to the LAS [29], we aimed to recruit at least 10 individuals for this group of neurologically intact adults.

First, we examined motor planning for unilateral shoulder abduction movement against gravity by using the presence of an SCM+ response. We tested the effect of trial type (voluntary, LAS_GO_), initial shoulder position (45°, 75° shoulder abduction), and their interaction on the probability of SCM+ using a binary logistic generalized linear mixed model with SCM+ responses set to 1, and longer or absent SCM responses set to 0. Participant was modeled as a random factor. We expected the probability of SCM+ to be greater in LAS_GO_ trials, consistent with startReact and indicating a motor action was prepared and triggered by the LAS. Next, we compared measures of motor initiation and execution by testing the effects of trial type (voluntary, LAS_GO_), initial position (45°, 75°), and their interaction on movement onset latency, movement duration, displacement, and peak velocity using linear mixed effects models with participant as a random factor and a restricted maximum likelihood solution. Only LAS_GO_ trials with evidence of early release of prepared motor action (SCM+) were included in this comparison. We hypothesized that movements triggered by the LAS would occur at shorter onset latencies but would not differ in movement duration, displacement and velocity compared to volitional movements. Finally, a separate linear mixed effects model was used to test the effect of muscle (DELT, left SCM, right SCM), trial type (voluntary, LAS_GO_), initial position (45°, 75°), and their interactions on muscle onset latency. Main effects and interactions were considered significant if p < 0.05. Significance level was adjusted using the Bonferroni correction for multiple pairwise comparisons. Estimated marginal means ± model standard errors are presented as measures of central tendency. P-values < 1e^-6^ are reported as p ~ 0; p-values < 0.001and ≥ 1e^-6^ are reported as p < 0.001; p-values > 0.95 are reported as p ~ 1.

### Control experiment

A secondary, control experiment was conducted to investigate the influence of our training protocol on variability in shoulder abduction displacement. Eight participants completed the control experiment (4 female, age: 22 ± 2 years). Two participants from the primary experiment returned and six additional participants were recruited using the same inclusion and exclusion criteria to complete this control experiment (**Table in** **S2 Table**). In the primary experiment, the target position was externally cued by the experimenter’s hand during the initial training trials and intermittently during testing based on displacement measured by the electrogoniometer. In the control experiment, the experimenter’s hand was replaced by a physical target, a foam rod, fixed at 30° displacement from the initial position. Our purpose was to provide continuous tactile feedback during training to reduce variability in movement displacement. The target was placed above the participant’s elbow until participants consistently responded rapidly to the GO cue and contacted the target at movement completion. Then the target was moved laterally but remained in the visual field to allow visual rather than tactile feedback during testing trials. All other details were the same in both experiments. During training for the control experiment with tactile feedback, an average of 37 ± 6 (range: 30–53) training trials were completed across participants and initial positions.

Methods for data processing and the exclusion of trials were identical to the primary experiment. For the control experiment, 4.4 ± 0.9% of trials across participants were excluded from analysis for protocol errors or no, early or late movement.

To test the hypothesis that tactile feedback during training would reduce variance in movement displacement during testing trials, we performed a one-sided F-test. The F-test compared the variances of movement displacement during voluntary trials between the primary and control experiments. We also tested the effect of tactile feedback during training on motor planning, initiation, and execution. First, the probability of SCM + was determined for the main effects of trial type and initial position and their interaction using a binary logistic generalized linear mixed model with only data from the control experiment. Then, to test the effect of tactile feedback during training on movement displacement and our other measures of motor initiation and execution, we used a linear mixed effects model for each dependent variable with participant as a random factor and a restricted maximum likelihood solution. Main effects of tactile feedback training condition (intermittent by the experimenter or continuous with physical target), trial type (voluntary, LAS_GO_), and initial position (45°, 75°) and their interactions were considered significant if p < 0.05. Data from both primary and control experiments were included.

## Results

### Primary experiment

Shoulder abduction angles are reported as angular displacements relative to the initial position to account for differences in absolute angles measured with the manual and electro gonimometers during calibration and differences in initial position across participants. Across participants, the shoulder abduction angle measured for the 45° initial position was 54 ± 5° (range: 45–59°) with the manual goniometer and 60.9 ± 6.7° (range: 47.1–70.1°) with the electrogoniometer. The angle measured across participants for the 75° initial position was 75 ± 1° (range: 75–80°) and 78.3 ± 9.8° (range: 63.7–97.0°) for the manual and electro goniometers, respectively. Across participants, average elbow displacement was 4.5 ± 2.3° during voluntary trials suggesting movement primarily occurred at the shoulder. No further analysis of elbow displacement was completed.

**Fig 2:**
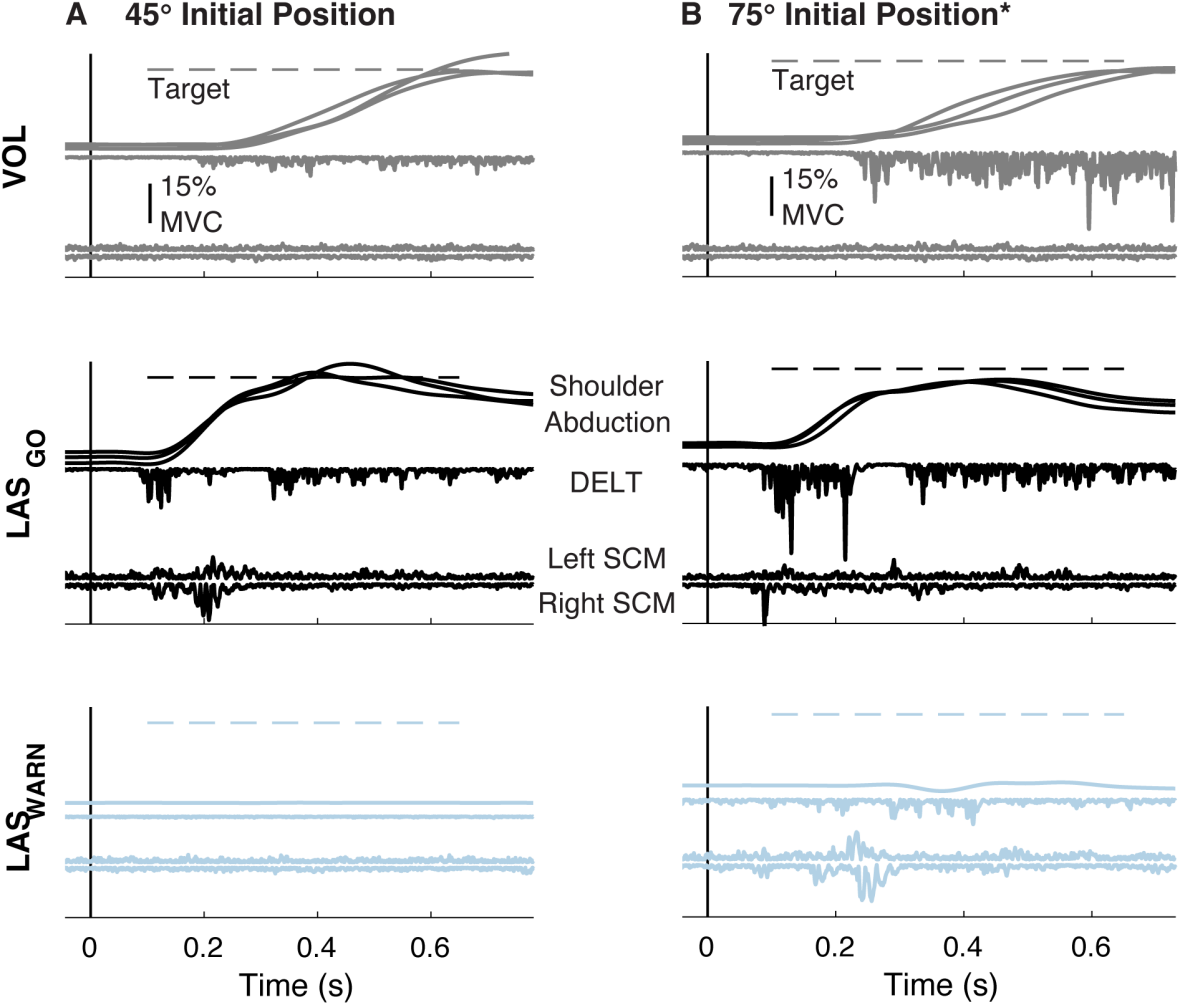
Exemplar data. Exemplary shoulder abduction, DELT, and SCM recordings during individual VOL, LAS_GO_ and LAS_WARN_ trials from the same participant. Initial position was varied to alter the influence of gravity at (**A**) 45° and (**B**) 75° abduction. The dotted line indicates the 30° shoulder abduction target relative to the initial position. All electromyography recordings are scaled to the 15% MVC bar (top row) with recordings ordered as indicated (middle row). Three trials are shown for VOL and LAS_GO_ shoulder abduction movement. A single trial is shown for LAS_WARN_ shoulder abduction movement. A single trial is shown for DELT and SCM recordings for each trial type. The exemplar LAS_GO_ trials shown here were classified as SCM + . *This participant completed the 75° initial position first. DELT: middle deltoid, GO: cue to move, LAS: loud acoustic stimulus, MVC: maximum voluntary contraction, SCM: sternocleidomastoid, s: seconds, VOL: voluntary, WARN: cue to prepare, °: degrees.

Shoulder abduction kinematics and EMG for a typical participant show that movement initiation was influenced by trial type for both initial shoulder abduction positions (Figs 2A and 2B). LAS_GO_ trials had shorter onset latencies than voluntary trials for both shoulder abduction movement and DELT muscle activation. Bilateral SCM muscle activation was elicited in LAS_GO_ trials at a short latency. Movement was directed towards the 30° target for both voluntarily initiated movements and those triggered in response to the LAS delivered at GO. LAS_WARN_ trials, in which the LAS was delivered prior to instructed motor preparation, did not elicit a sustained DELT response or substantial displacement from the initial position.

**Fig 3:**
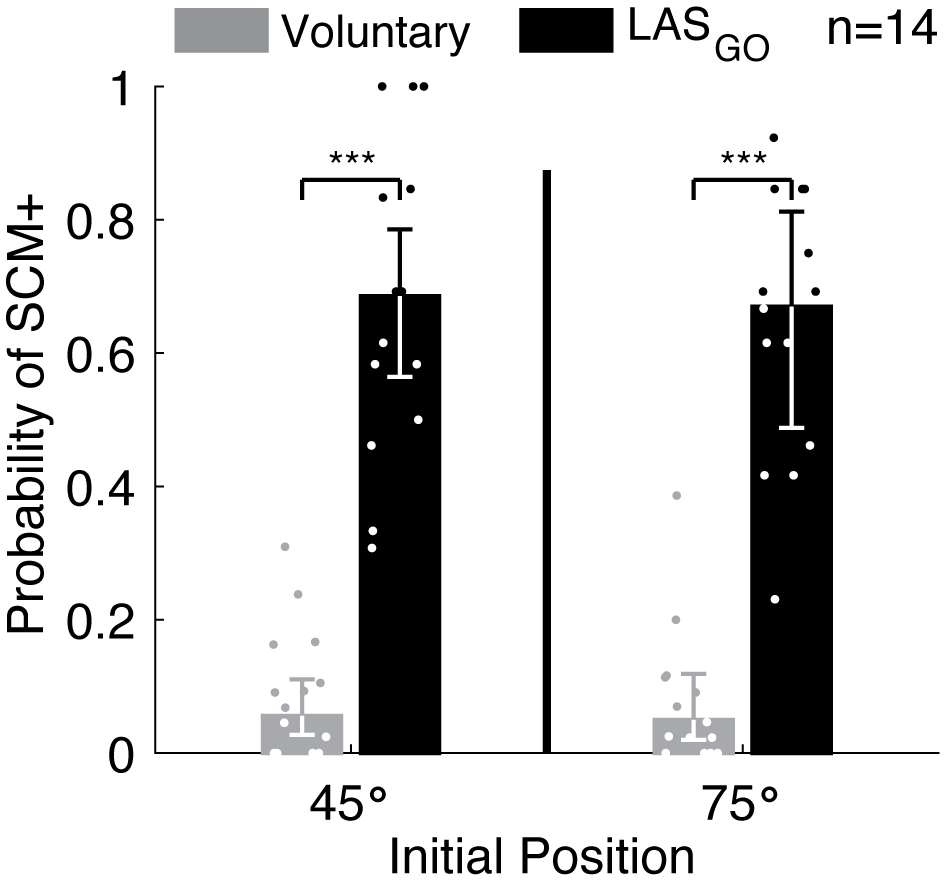
Probability of eliciting early sternocleidomastoid (SCM) activity. Group results for the influence of trial type and initial position on the probability of an SCM+ response. Group mean ± standard error reported from linear mixed effects model. Individual participant probabilities are shown as dots. Significant pairwise comparisons are indicated for p < 0.001 (***). GO: cue to move, LAS: loud acoustic stimulus, n: number of participants, SCM + : rapid sternocleidomastoid onset < 120 milliseconds, °: degrees.

The LAS delivered at the GO cue was more likely to elicit early SCM muscle activation (SCM+) than voluntary movement initiation (Fig 3). The probability of SCM+ differed with trial type (Wald χ^2^ = 374, p ~ 0) but not initial position (Wald χ^2^ = 1.2, p = 0.28) or the interaction (Wald χ^2^ ~ 0, p ~ 1). Averaged across initial position, the probability of SCM+ for LAS_GO_ trials was greater (0.68 ± 0.05, 95% confidence interval = [0.58, 0.77]) than the probability of SCM+ for voluntary trials (0.07 ± 0.01, 95% confidence interval = [0.05–0.10], p < 0.001).

The LAS was also delivered at the WARN cue when we would not expect participants to have prepared a motor plan. SCM+ responses were observed for ~25% of LAS_WARN_ trials. However, evidence of a prepared motor action was apparent in only one of these trials with bilateral SCM activation at ~60 ms and >20° displacement towards the target. The remaining LAS_WARN_ trials with SCM+ responses were not consistent with motor planning. These trials either had delayed shoulder abduction movement onset latencies (>267 ms), suggesting voluntary initiation, or had displacement <1°, suggesting a startle response occurred in the absence of a prepared motor action. LAS_WARN_ trials were excluded from further analysis.

**Fig 4:**
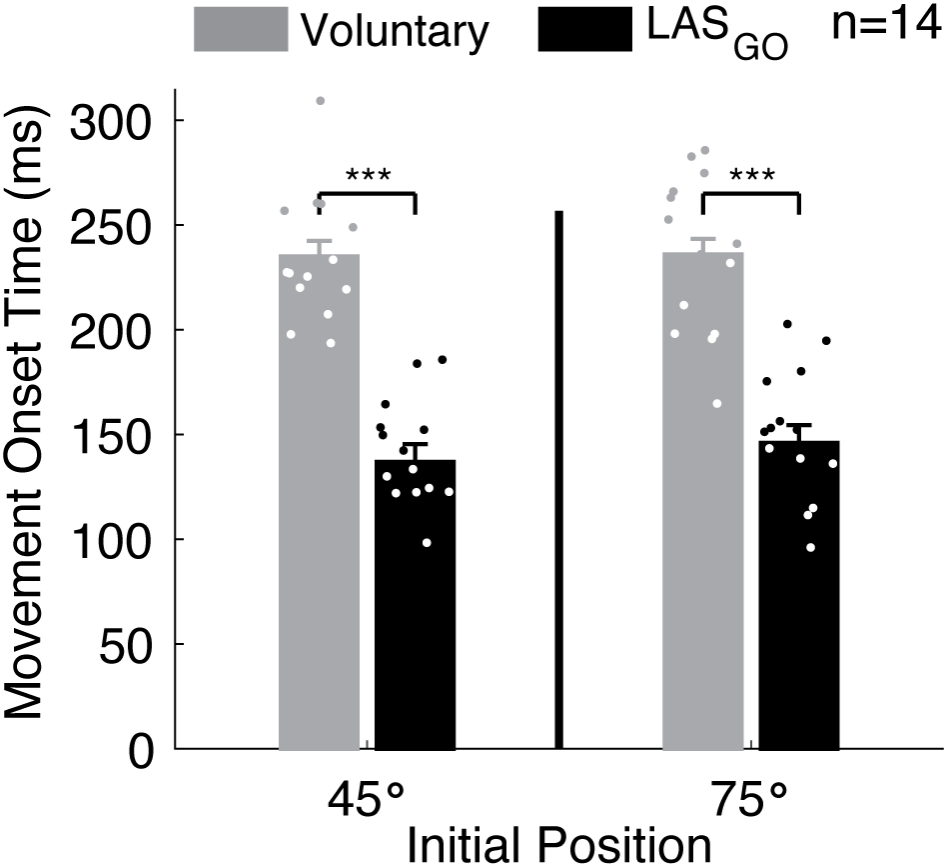
Movement onset latency. Group results for the influence of trial type and initial position on movement onset latency. Group mean ± standard error reported from linear mixed effects model. Individual participant means are shown as dots. Significant pairwise comparisons indicated for p < 0.001 (***). GO: cue to move, LAS: loud acoustic stimulus, ms: milliseconds, n: number of participants, °: degrees.

Motor initiation differed for movements initiated voluntarily and those triggered by the LAS_GO_. Across all participants, the onset latency of shoulder abduction movement was influenced by trial type (Wald χ^2^ = 674, p ~ 0) but not initial position (Wald χ^2^ = 1.7, p = 0.19) or the interaction (Wald χ^2^ = 1.2, p = 0.28) (**Fig 4**). Averaged across initial position, movement occurred at a shorter latency when triggered in response to the LAS_GO_ (142 ± 8 ms) than when initiated voluntarily (235 ± 7 ms, Δ = 94 ± 4 ms, p < 0.001).

**Fig 5:**
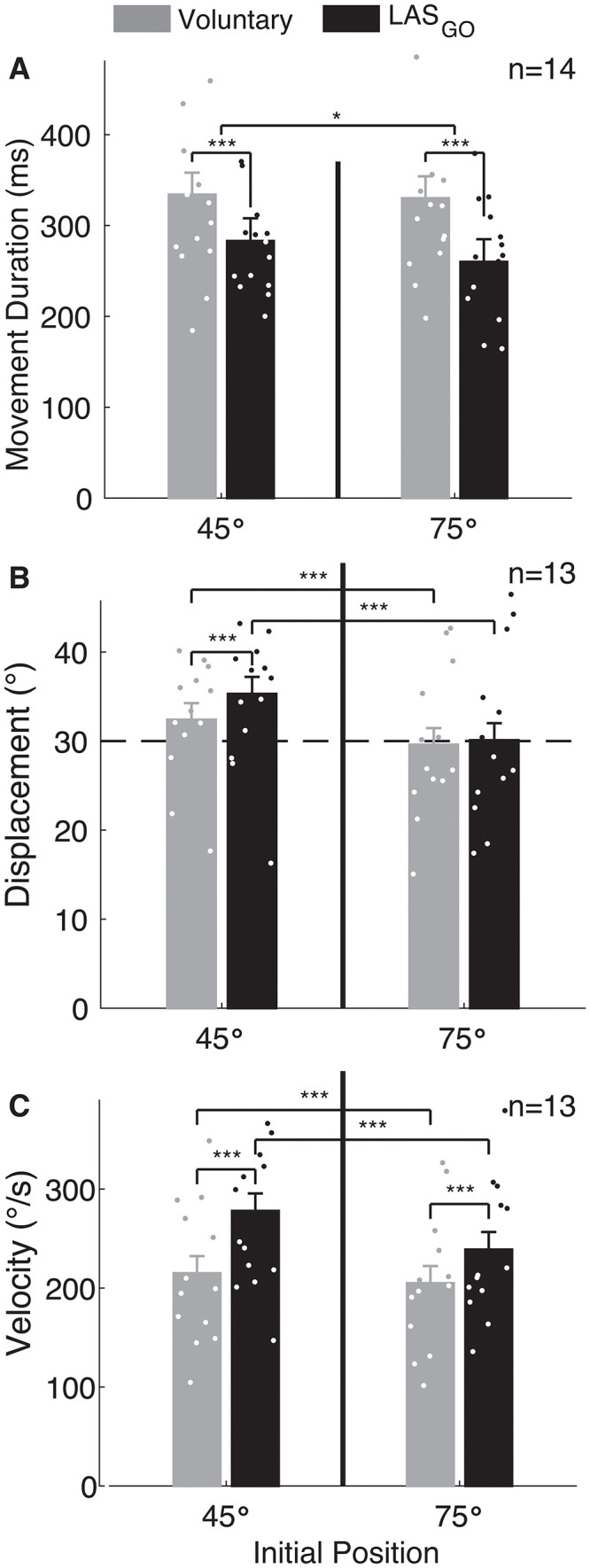
Movement duration, displacement and peak velocity. Group results for the influence of trial type and initial position on shoulder abduction **(A)** movement duration, **(B)** displacement and **(C)** peak velocity. Group mean ± standard error reported from linear mixed effects model. Individual participant means are shown as dots. Significant pairwise comparisons indicated for p < 0.05 (*) and p < 0.001 (***). GO: cue to move, LAS: loud acoustic stimulus, ms: milliseconds, n: number of participants, s: seconds, °: degrees.

Unexpectedly, we also found that the execution of shoulder abduction movement differed for movements initiated voluntarily and those triggered by the LAS_GO_. First, movement duration was influenced by trial type (Wald χ^2^ = 120, p ~ 0) and initial position (Wald χ^2^ = 5.7, p = 0.017) but not the interaction (Wald χ^2^ = 3.0, p = 0.08) (**Fig 5A**). Averaged across initial positions, movement duration was shorter when triggered in response to the LAS_GO_ than when initiated voluntarily (Δ = 61 ± 6 ms, p < 0.001). Averaged across trial type, movement duration was longer for the 45° than 75° initial position (Δ = 13 ± 6 ms, p = 0.018). Second, movement displacement was influenced by trial type (Wald χ^2^ = 17.9, p < 0.001), initial position (Wald χ^2^ = 110, p ~ 0), and the interaction (Wald χ^2^ = 8.8, p = 0.003) (**Fig 5B**). For the 45° initial position, displacement was greater when triggered in response to the LAS_GO_ than when initiated voluntarily (Δ = 2.7 ± 0.5°, p < 0.001), but trial type did not influence displacement for the 75° initial position (p = 0.37). Displacement was greater for the 45° initial position than the 75° initial position for both voluntary (Δ = 2.9 ± 0.3°, p < 0.001) and LAS_GO_ trials (Δ = 5.1 ± 0.7°, p < 0.001). Third, peak movement velocity was influenced by trial type (Wald χ^2^ = 155), initial position (Wald χ^2^ = 43), and the interaction (Wald χ^2^ = 14.0, all p < 0.001) (**Fig 5C**). Movements triggered in response to the LAS_GO_ had a faster peak velocity than voluntary trials for 45° (Δ = 57 ± 5°/s, p < 0.001) and 75° (Δ = 31 ± 5°/s, p < 0.001) initial positions. Peak velocity was significantly faster for the 45° initial position than the 75° initial position for voluntary (Δ = 10 ± 3°/s, p < 0.001) and LAS_GO_ (Δ = 36 ± 6°/s, p < 0.001) trials.

**Fig 6:**
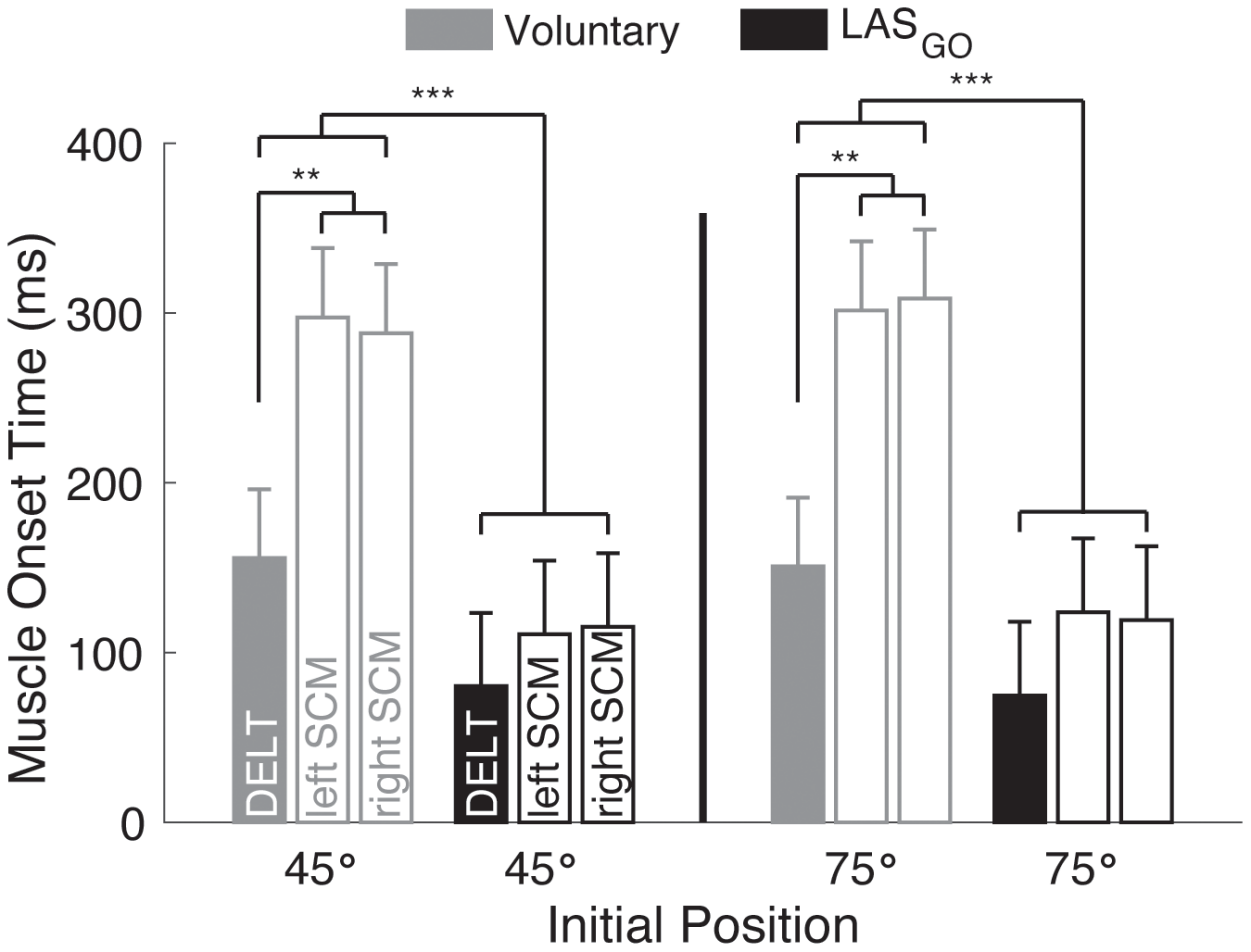
Muscle onset latency. Group results for the influence of muscle, trial type, and initial position on the onset latency of DELT, left SCM, and right SCM. Group mean ± standard error reported from linear mixed effects model. Significant pairwise comparisons indicated for p < 0.001 (***) and p < 0.01 (**). DELT: middle deltoid, GO: cue to move, LAS: loud acoustic stimulus, ms: milliseconds, SCM: sternocleidomastoid, °: angular degrees.

The onset latencies and relative timing of the primary agonist (DELT) and SCM muscles differed for movements initiated voluntarily and those triggered by the LAS_GO_. Muscle onset latencies were influenced by trial type (Wald χ^2^ = 343, p ~ 0) and the interaction between muscle and trial type (Wald χ^2^ = 41, p ~ 0) but not muscle (Wald χ^2^ = 5.4, p = 0.067), initial position (Wald χ^2^ = 0.4, p = 0.5), or interactions with initial position (all Wald χ^2^ < 0.9, all p > 0.6) (**Fig 6**). Averaged across muscles and initial position, muscle onset latencies were shorter in response to the LAS_GO_ compared to movements initiated voluntarily (all p < 0.001). For voluntary trials, the onset latency of the DELT (153 ± 40 ms) was earlier than the onset latencies of the left SCM (Δ = 146 ± 46 ms, p = 0.0014) and right SCM (Δ = 145 ± 46 ms, p = 0.0015) muscles. For LAS_GO_ trials, there was no difference in onset latency for the DELT (78 ± 42 ms) and SCM muscles (Δ = 40 ± 48 ms, both p > 0.41). There was no difference in the muscle onset latency of the left and right SCM for voluntary or LAS_GO_ trials (both p ~ 1).

### Control experiment

Tactile feedback during training reduced variance in movement displacement during voluntary testing trials (F = 1.8, p ~ 0), as was intended. In addition, the interpretation of key findings for motor planning and initiation of shoulder abduction movement were not influenced by tactile feedback. Specifically, when tactile feedback was provided, there remained a significantly greater probability of SCM+ response for LAS_GO_ trials than voluntary trials, indicating motor planning was observed in both experiments. There also remained a significant decrease in movement and muscle onset latency for LAS_GO_ compared to voluntary trials; though, tactile feedback did significantly reduce movement onset latency for voluntary trials (Δ = 43 ± 17 ms, p = 0.017).

The addition of tactile feedback during training reduced the difference in displacement between voluntary movements and those triggered in response to the LAS delivered at GO that was observed for the 45° initial position (Fig 5B). In contrast to the primary experiment, with tactile feedback there was no significant difference in displacement between LAS_GO_ and voluntary trials for either initial position (45°: Δ = 0.2 ± 0.6°, 75°: Δ = 0.2 ± 0.6°, both p > 0.75). All other conclusions for motor execution measures from the primary experiment were unchanged when tactile feedback was used.

## Discussion

This study investigated motor planning, initiation and execution of unilateral shoulder abduction. We studied the planning phase of motor control independent of the initiation and execution phases using StartReact. Our results have demonstrated that planned unilateral shoulder abduction movements against gravity can be triggered involuntarily by a LAS. For two initial positions, we found that the initiation of LAS-triggered movements occurred with shorter onset latencies of both the DELT muscle and shoulder abduction movement compared to those initiated voluntarily, consistent with prior startReact studies [16,18,19,21,29,39–41,43]. Movements triggered by the LAS were shorter in movement duration and faster in peak velocity than those initiated voluntarily. When tactile feedback was provided during training, movement displacement did not differ between movements triggered by the LAS and those initiated voluntarily. These results suggest altering the influence of gravity on unilateral shoulder abduction movements in young adults without neurological deficits did not influence measurements of motor planning and can inform further research to understand deficits in startReact reported in the literature following stroke.

### StartReact can be used to investigate the contributions of task requirements and stroke to motor planning

Our results are important for understanding the contributions of motor planning to impairments in the initiation and execution of reaching following stroke. The presence of startReact responses following stroke has been used to suggest motor planning is intact for movements of the distal upper extremity including elbow flexion, elbow extension, and hand opening, even though deficits are evident with voluntary movement initiation and execution [19,21]. However, evidence of intact motor planning has been limited in startReact studies post stroke with tasks requiring shoulder abduction [22–24]. For instance, a low probability of LAS-triggered movement has been reported as evidence of impaired motor planning for isometric shoulder abduction during elbow movement [24] and forward reaching [22,23]. Our results demonstrate startReact responses are intact for unilateral shoulder abduction movement against gravity in individuals without history of stroke. A reasonable next step is applying this paradigm to investigate isolated shoulder movement following stroke. If LAS-triggered movements are absent or impaired for an isolated shoulder task, then stroke may limit motor planning of shoulder abduction movement against gravity. In contrast, if LAS-triggered movements are present for an isolated shoulder task, as in this study, then additional task requirements for multi-joint coordination or postural maintenance may contribute to evidence of impaired motor planning reported in prior studies. Understanding the limitations of using startReact to trigger a prepared motor action for proximal upper extremity movements post stroke will inform the therapeutic potential of startReact as a mechanism for rehabilitation.

### Kinematic similarity of voluntary and LAS-triggered movements

One defining characteristic of startReact to date has been similar kinematics for both voluntary and LAS-triggered movements [16,17]; however, in our primary experiment we found differences in shoulder abduction displacement and velocity across these trial types. Our rationale for expecting similar movement kinematics across trial types is the premise that the same prepared motor action is observed whether initiated voluntarily or triggered involuntarily by a LAS via neural circuits that overlap with the startle reflex [17,18,39,44]. However, experimental support for this has been variable and limited. While some studies have reported that the magnitude of displacement was maintained between voluntary and startReact movements [18,39], others reported a significant difference [29,45] or did not quantitatively report displacement [19,21,41,43,46]. Even fewer studies have reported velocity for voluntary and LAS-triggered movements. One study found no difference [39] while another found larger peak velocities during startReact movements [45]; several other studies did not quantify or report movement velocity for both voluntary and startReact movements [19,21,29,40,44]. In our control experiment, shoulder abduction displacement did not differ between voluntary and LAS-triggered movements while peak velocity remained faster for LAS-triggered movements highlighting the importance of task training in startReact studies. Given the variable findings and lack of experimental data reported in the literature, additional research is needed to determine if kinematic similarity between voluntary and LAS-triggered movement is in fact a defining characteristic of motor planning observed using startReact.

### Considerations for applying startReact methodology to study motor control of the shoulder

The fact that the shoulder is a complex of multiple joints and moves in multiple planes presents unique challenges that must be considered when studying its motor control. We specifically designed our task for isolated shoulder abduction to limit the confounding factors of multi-joint or postural tasks. However, doing so resulted in use of the elbow as the end effector. Since the elbow is not typically a functional endpoint, we did not provide visual feedback of elbow position. In contrast, previous startReact studies have used the hand or wrist as a natural endpoint for upper extremity movement and provided direct or computerized feedback to visualize position of the end effector [19,20,29–31,39,40,44]. In our primary experiment, we found that LAS-triggered movements had greater displacement than voluntary movements only when initiated from 45° of shoulder abduction and hypothesized this was due to increased movement variability. Increased variability in movement displacement could result from low prioritization of endpoint accuracy for the elbow and/or lack of external feedback. If elbow endpoint position was not prioritized as a component of the motor plan, we hypothesized that faster, LAS-triggered response could result in overshooting the target from the 45° initial position in which the range of motion was not limited biomechanically (as it was from 75°) [47]. To test this in a control experiment, we added tactile sensory feedback of the target position to increase prioritization of endpoint accuracy and found that movement displacement no longer differed between LAS-triggered and voluntary movement. While the different participant groups for the primary and control experiments could have influenced these findings, we suggest that training, including the type of feedback and amount of training provided, is critical to guide motor planning and selection of key measures in startReact studies.

Control of the shoulder differs from other upper extremity joints due to its large range of motion and greater requirements for postural maintenance and movement against gravity. Previous startReact studies at the elbow or wrist have used equipment and positioning to minimize the effects of gravity and restrict movement to a single degree of freedom [19,20,29,30,39,40,44–46]. For example, straps have been used to restrict trunk movement, minimizing the effort required for postural control and focusing motor planning demands on a single distal upper extremity joint [19,29,39,40,44,45]. However, to study the shoulder we chose not to restrict the trunk to allow for scapulothoracic movement, which is essential for abduction, thereby, introducing additional requirements for motor planning, specifically the maintenance of trunk posture and scapulothoracic movement in addition to glenohumeral abduction. We also studied two initial positions to vary the effect of gravity and joint mechanics on the 30° shoulder abduction movement. Due to gravity, more torque was required to complete the task from 75° than from 45° abduction. However, the effect of gravity may have been confounded by the portion of the range of motion in which movement occurred. Movement initiated from 75° abduction was likely terminated near the joint’s end range given that the shoulder was internally rotated, limiting subacromial space with humeral elevation above 60°. In contrast, movement initiated from 45° abduction occurred in mid-range, similar to startReact studies of distal upper extremity movements [19,29,30,39–41,44,47]. We suggest that, rather than an effect of gravity, the biomechanical limitation of movement initiated from 75° abduction contributed to more consistent movement displacement even without external sensory feedback of elbow position.

We found that delivery of a LAS elicited movement with shorter onset latencies in the DELT muscle, the prime mover of glenohumeral joint abduction, consistent with startReact studies of the fingers [48,49], wrist [29,40,44], elbow [18,19,39,41,43], and bilateral shoulders [25]. However, the motor control of the shoulder complex cannot be fully described by prime movers or agonist and antagonist muscle pairs as has been typical in studies of uniplanar movements of distal upper extremity joints. Instead, control of the shoulder complex relies on the coordinated action of muscles across multiple joints, including scapulothoracic and glenohumeral muscles [1,28], to achieve proximal stability and humeral elevation. Due to its relatively limited biomechanical stability and complex joint and muscle anatomy, impairments in motor control are especially impactful at the shoulder [50–53]. Therefore, recommendations for shoulder rehabilitation emphasize the assessment and intervention of motor control [54–60]. These recommendations focus primarily on the execution phase of motor control, as there is far less known about the motor planning of shoulder movements. Future studies could use startReact to assess motor planning and initiation of glenohumeral and scapulothoracic muscle coordination during movement, adding to our knowledge of motor control during execution and current rehabilitation recommendations.

Finally, we used SCM as an independent indicator of startle; however, we also observed SCM muscle activation during volitional movement for some participants, limiting exclusion of voluntary trials with SCM+ response. This is consistent with studies of ballistic neck movement [61] and postural perturbations applied to the arm [62], where the SCM had a role in volitional movement, either as a prime mover or to stabilize the head and neck. The SCM muscle spans the shoulder complex so, unlike at distal upper extremity joints, it can contribute to voluntary movement of the shoulder. Thus, it is possible that SCM muscle activity during LAS_GO_ trials resulted from a rapid voluntary response and was not triggered by the startle response. In addition, ballistic movement of the proximal upper extremity may require bilateral coordination of neck muscles, possibly including the SCM, to stabilize the head. We found that the muscle onset latency of the left and right SCM muscles did not differ for voluntary trials; however, it is not clear if this supports its role as a prime mover, in stabilizing the head, or both. Consistent with our methods, other startReact studies that recorded from the SCM muscle bilaterally used the activation latency of either the left or right muscle to indicate an SCM+ response [19,24,25]. Indeed, recent work has quantified a bilaterally asymmetric influence of posture on the latency of SCM muscle activation for shoulder abduction [63]. Characterization of the role of the SCM during movement of the shoulder complex could inform future startReact protocols.

Prior studies have compared muscle onset latencies between SCM+ and SCM- responses to the LAS to distinguish an influence of stimulus intensity from startReact [25,49]. Despite using a lower intensity LAS (114 dB), the probability of SCM+ measured across the group of participants remained high (~0.70). Only seven participants in our primary experiment had a probability of SCM+ between 0.30 and 0.70 for LAS_GO_ trials. We did not feel this was adequate data for a comparison of SCM+ and SCM- trials as a primary result. We did perform a preliminary analysis of these participants and found movement occurred at a shorter onset latency for both LAS_GO_ trial types compared to voluntary trials (233 ± 9 ms, both p < 0.001) and for LAS_GO_ trials with SCM+ response (149 ± 10 ms) compared to SCM- response (189 ± 10 ms, p < 0.001). The results of this preliminary SCM + /- analysis are consistent with a recent study of bilateral shoulder abduction suggesting reticulospinal drive to the shoulder musculature facilitates a robust startReact effect [25]. Though our analysis is consistent with prior studies classifying startReact by comparing SCM onset latency to a cutoff [17,19,24,26,38,40,41,64], the presence of SCM muscle activation during volitional movement warrants further investigation to determine the robustness of this classification.

### Significance

Physical therapy interventions for upper extremity movement following stroke generally emphasize strength, range of motion, and coordination with current knowledge of motor control impairments primarily limited to the execution phase of movement. Our study used startReact to investigate motor planning, initiation and execution of shoulder abduction from two initial positions to investigate the influence of gravity in adults without a history of stroke. Our results demonstrated that motor planning of unilateral shoulder abduction was not influenced by gravity throughout the range of motion. We found that differences in the execution of the prepared motor action when triggered using startReact depended on task training, not initial position. These findings provide further evidence that sensorimotor control of the shoulder complex can be investigated using startReact, including the influence of gravity and task requirements, to understand impairments in motor planning that may result from stroke. This work is important to guide studies quantifying deficits in shoulder control for individuals with neurological disease or injury and to develop effective, phase-specific interventions with the potential to improve rehabilitation.

## Supporting information

S1 Table Summary of primary experiment participant demographics, preliminary measures, and manual goniometry. The smallest achievable shoulder angle was measured with a manual goniometer. Participants did not report race or ethnicity. cm: centimeters, F: female, M: male, OWD: occiput-to-wall distance, y: years, **°**: angular degrees.
(DOCX)

S2 Table Summary of control experiment participant demographics, preliminary measures, and manual goniometry. The smallest achievable shoulder angle was measured with a manual goniometer. Participants did not report race or ethnicity. cm: centimeters, F: female, M: male, OWD: occiput-to-wall distance, y: years, °: angular degrees.
(DOCX)

S3 Dataset Processed trial data included for the primary and control experiments. Participant: unique identifier; Trial_Type: 10: voluntary, 11: LAS_WARN_, 12: LAS_GO_; Initial_Position: 45: 45° initial position, 75: 75° initial position; Tactile_Feedback: 0: primary experiment, 1: control experiment; Onset: shoulder abduction onset, Offset: shoulder abduction offset, Movement Time: movement completion time; Displacement: shoulder abduction displacement: Velocity: peak shoulder abduction velocity; RSCM_Onset: muscle onset latency of right SCM muscle; LSCM_Onset: muscle onset latency of left SCM muscle; Delt_Onset: muscle onset latency of deltoid muscle; SCM_Classification: 0: SCM-, 1: SCM+.
(CSV)
