# Supplementary material for: Motor planning, initiation and execution of shoulder abduction against gravity: Evidence from startReact
Source: PLoS One. 2026 Apr 10;21(4):e0346615. doi: 10.1371/journal.pone.0346615 (PMC13068219; doi:10.1371/journal.pone.0346615)
Supplement: S2 Table — The smallest achievable shoulder angle was measured with a manual goniometer. Participants did not report race or ethnicity. cm: centimeters, F: female, M: male, OWD: occiput-to-wall distance, y: years, °: angular degrees. (DOCX) [file pone.0346615.s002.docx]

**S2 Table.** **Summary of control experiment participant demographics, preliminary measures, and manual goniometry.**

| **Age (y)** | **Sex** | **OWD (cm)** | **Smallest Achievable Shoulder Angle for 45º Initial Position (°)** |
| --- | --- | --- | --- |
| 19 | F | 3.00 | 55 |
| 20 | F | 4.33 | 55 |
| 20 | M | 4.80 | 45 |
| 21 | M | 3.67 | 60 |
| 22 | M | 6.00 | 60 |
| 23 | F | 4.33 | 45 |
| 24 | F | 5.00 | 58 |
| 24 | M | 3.00 | 50 |

The smallest achievable shoulder angle was measured with a manual goniometer. Participants did not report race or ethnicity.

cm: centimeters, F: female, M: male, OWD: occiput-to-wall distance, y: years, **°**: angular degrees
